# Determinants of Enrolment and Renewing of Community-Based Health Insurance in Households With Under-5 Children in Rural South-Western Uganda

**DOI:** 10.15171/ijhpm.2019.49

**Published:** 2019-07-06

**Authors:** Emmanuel Nshakira-Rukundo, Essa Chanie Mussa, Nathan Nshakira, Nicolas Gerber, Joachim von Braun

**Affiliations:** ^1^Institute for Food and Resource Economics (ILR), University of Bonn, Bonn, Germany.; ^2^Department of Economics and Technological Change, Center for Development Research (ZEF), University of Bonn, Bonn, Germany.; ^3^Department of Environmental and Public Health, Kabale University, Kabale, Uganda.

**Keywords:** Community-Based Health Insurance, Enrolment, Renewing, Perceptions, Rural Uganda

## Abstract

**Background:** The desire for universal health coverage in developing countries has brought attention to communitybased health insurance (CBHI) schemes in developing countries. The government of Uganda is currently debating policy for the national health insurance programme, targeting the integration of existing CBHI schemes into a larger national risk pool. However, while enrolment has been largely studied in other countries, it remains a generally under-covered issue from a Ugandan perspective. Using a large CBHI scheme, this study, therefore, aims at shedding more light on the determinants of households’ decisions to enrol and renew membership in these schemes.

**Methods:** We collected household data from 464 households in 14 villages served by a large CBHI scheme in southwestern Uganda. We then estimated logistic and zero-inflated negative binomial (ZINB) regressions to understand the determinants of enrolment and renewing membership in CBHI, respectively.

**Results:** Results revealed that household’s socioeconomic status, husband’s employment in rural casual work (odds ratio [OR]: 2.581, CI: 1.104-6.032) and knowledge of health insurance premiums (OR: 17.072, CI: 7.027-41.477) were significant predictors of enrolment. Social capital and connectivity, assessed by the number of voluntary groups a household belonged to, was also positively associated with CBHI participation (OR: 5.664, CI: 2.927-10.963). More positive perceptions on insurance (OR: 2.991, CI: 1.273-7.029), access to information were also associated with enrolment and renewing among others. Burial group size and number of burial groups in a village, were all significantly associated with increased the likelihood of renewing CBHI.

**Conclusion:** While socioeconomic factors remain important predictors of participation in insurance, mechanisms to promote inclusion should be devised. Improving the participation of communities can enhance trust in insurance and eventual coverage. Moreover, for households already insured, access to correct information and strengthening their social network information pathways enhances their chances of renewing.

## Background

The 2010 World Health Report suggested that apart from the availability and equitable use of resources, reliance on direct payments for health was another barrier to universal health coverage, leading to increased catastrophic health expenditures.^1^ Xu et al^2^ estimated that about 150 million people faced catastrophic health expenditures and 100 million people were pushed into poverty annually due to catastrophic health payments. Moreover, poor households are more likely to borrow and or sell their household productive assets when faced with such health payments.^3^ To protect households from eminent deprivation due to health expenditures and encouraging policies for universal health coverage, the World Health Organization (WHO) has recognised the role of community-based health insurance (CBHI) schemes. In addition to previous pronouncements in support of CBHI (such as Resolution 58.33 of the 2005 World Health Assembly),^4^ the 2010 WHO has stated that countries need “longer-term plans for expanding prepayment and incorporating community and micro-insurance into the broader pool” including “voluntary schemes, such as community health insurance or micro-insurance….”^1^

The contributions of CBHI schemes to health systems financing and broader pathways to universal health coverage in developing countries are well-documented.^5,6^ In detail, substantial research has explored questions of enrolment^7-9^ and renewing.^10^ Nonetheless, in Uganda, a country with a long history of CBHI, very little is known about these questions, especially from a quantitative perspective. Previous work has only used qualitative methods, identifying various issues such as lack of trust, limited understanding from both policy-makers and clients, and limited community involvement, among the factors inhibiting enrolment.^11-13^ Only 3 studies try to address these questions quantitatively. Biggeri et al^14^ study the feasibility of CBHI in a region without prior experience in a willingness to pay exercise. Cecchi et al,^15^ using a public good experiment, study the dynamics of social capital when third-party – run CBHI is introduced in villages. They reveal that social capital suffers when insurance is formalised through CBHI schemes. None of these studies directly addresses the main questions, why households join and why households remain in CBHI schemes. The closest to our study is^16^ who use mixed methods to investigate why rural households choose to enrol in insurance over free health services. This study is also in a similar area like ours. They find that overall poor quality services, drug stock-outs as well as poor human resourcing pushed households from free government health services while easier access to healthcare, financial protection, the perception of the quality of care and the intrinsic benefits of mutual assistance attracted individuals to CBHI. Our main objective here is to contribute to this body of work by directly addressing these questions.

Overall, the literature on enrolment, as elaborated in several systematic reviews^7-9,17^ can be summarised in 2 dimensions. Firstly, the legal, institutional and policy environment in which insurance operates is important.^18-20^ Countries with stronger laws also have the political will to facilitate higher enrolment. However, major bottlenecks to voluntary enrolment are associated with households’ socioeconomic capacities to demand. Wealthier, better educated^7,21^ and people with positive perceptions about insurance^22^ and more informed individuals^10^ are more likely to enrol. Moreover, specific groups such as women in reproductive age^23^ and children^24^ face distinct barriers to enrolment in comparison to the general population.

While enrolling in insurance remains of pertinent interest, dropping out of insurance is high.^25-27^ A handful of papers have looked into this issue.^10,28,29^ The other purpose of this research is to add to this thin literature. Moreover, for Uganda, this analysis is of further policy interest. After many years of a slow policy process,^30^ the government is in the process of starting a national health insurance scheme. The scheme will aim to build on and integrate existing community insurance schemes into a larger risk pool. These results will, therefore, feed into the policy process, in a timely fashion, to give a better understanding of what influences rural households’ decisions to participate and renew participation.

### The Landscape of Health Insurance in Uganda

Uganda does not have any public insurance programme. The current health financing policy provides that general health services are free at public health facilities.^31^ Private non-profit health facilities receive grants to subsidise services but also charge user fees.^32,33^ However, there has been a long-standing process of starting a public health insurance programme.^30^ Coverage of private insurance schemes is lean, available mainly to urban formal sector employed individuals and estimated at only about 460 000 people in 2012.^34^ CBHI is, therefore, the remaining option for rural, informal and poor households. Musau^35^ profiled the first CBHI scheme in Uganda, the Kisiizi Hospital CBHI scheme, and since then, the schemes have grown to 21 schemes covering over 140 000 people in 2014.^36^ The schemes are mainly in central and western Uganda, especially in regions that have been known to have burial societies which provide informal mutual insurance.^37^ While previous studies have shown a low demand for CBHI in Uganda,^11,12^ recent studies have shown increasing interest.^14,15^ It is understood that in the financial year 2018/2019, the revised National Health Insurance Bill will be approved into law for the establishment of a national health insurance scheme.^38^

### The Kisiizi Hospital Community-Based Health Insurance Scheme

The Kisiizi Hospital CBHI scheme is the largest CBHI scheme in Uganda, providing insurance coverage to over 42 000 individuals. Households pay premiums ranging from UGX (Uganda Shilling; Uganda currency) 11 000 per person for a household of 8-11 members to UGX 28 000 per person for a 2-person household. In US dollar terms, at the time of data collection in August 2015, this was equivalent to US$3 for 8-11 member household and US$8 for the 2 person-households. Accordingly, these premiums were equivalent to 1%-2% of the annual income in south-western Uganda in 2015.^39^ Enrolment in insurance is group based in that households that participate organise themselves in groups. However, the scheme does not operate as group insurance since there is no joint liability in the group. Majority of the groups (about 95%) were traditional funeral groups which have existed in the area for very many decades.^37^ However, the unit of enrolment is a household and groups are only used as marketing and coordination platforms. Currently, about 210 groups belong to the scheme. Households are required to enrol as a full unit and not partial enrolment as observed in other schemes such as in Ghana.^40^ An important feature of the scheme is the waiting time for full coverage. Newly enrolled households typically wait for about 12 months to be fully covered. Newly enrolled members pay 90% of the medical costs when they are hospitalised within the first 12 months of enrolment. This waiting time is significantly longer than what is observed in other schemes such as in Nigeria.^41^ These conditions are aimed to control moral hazard. The scheme covers basic primary care, maternity care, surgeries, and outpatient and inpatient services and excludes outpatient services for chronic illnesses and substance abuse related illnesses and injuries.

## Methods

### The Data

Data used in this study comes from a cross-sectional survey conducted between August and December 2015, in Kabale and Rukungiri districts in south-western Uganda. A multi-stage simple random sampling criterion was applied to select a population representative sample of 464 households in 14 villages. The first stage was the selection of villages from 3 sub-counties of Nyakishenyi and Nyarushanje in Rukungiri district and Kashambya sub-county in Kabale district, which have the highest coverage of Kisiizi CBHI scheme. The 3 sub-counties represented a population of 106 000 people in 23 500 households as of the 2014 national census.^42^ We invited leaders from 23 parishes in the 3 sub-counties for a first stage sampling workshop. Fifteen of the 23 parish leaders attended in person or were represented by a committee member. Eight parishes that did not have a representative were excluded. All parish leaders were requested to list all the villages in their area. In addition, they were requested to classify the villages into rich and poor, using access to road, school or health facility or market as a criterion. Altogether, 174 villages were listed, 104 as poor and 70 as rich villages. All the listed villages’ names were put in a raffle box according to their categorisation and a leader randomly selected 7 villages from each box in the presence of other leaders and the research team. Leaders who attended the village sampling workshop provided the contacts of lower level leaders in the selected villages for household listing.

The second stage of sampling was household listing and selection of households for the survey. Fourteen lower level leaders were invited for a household listing workshop and requested to generate a list of households in their villages who had a child between 6 months and less than 59 months (5 years) . A total of 511 households were listed and 464 were interviewed.

A data collection tool was developed by the first and fourth authors and was duly assessed by the respective ethical committees in Germany and Uganda. The tool included a household demographic module collecting data on household occupancy; a child and maternal health module recording data on healthcare seeking behaviour for mothers and children and a nutrition module recording household food availability and intake data. Data on durable assets holdings and other endowments in agriculture, water and sanitation, and housing was recorded as an indicator for household social and economic welfare. The health insurance and social connectivity modules collected data regarding household insurance status, group membership and participation, and knowledge of insurance such as premiums and benefits package. In line with,^22^ data on various perceptions on insurance were collected. Moreover, village level information is also collected and used to control for village heterogeneity.

Data were collected using Open Data Kit, a computer-assisted personal interviewing platform. Open Data Kit and other platforms of similar fashion are becoming increasingly suggested for their overall cost-effectiveness and reducing of common survey errors.^43^ Data analysis was conducted in Stata version 14.^44^

### Empirical Approach

We employ 2 models to understand the determinants of enrolment and renewing CBHI. Since the outcome for CBHI participation (1 if CBHI member and 0 otherwise), the suitable model is a binary logistic model to estimate the determinants of household’s CBHI status. The model is given as:

Pr (*Insure*=1)_i_ = *β*_0_*+β*_1_*X*_1i_*+ β*_2_*X*_2i_*+β*_3_*X*_3i_*+ϵ*_i_

Where the probability that a household *i* was enrolled depends on *X*_1i_ – a vector of household socioeconomic and demographic variables, *X*_2i_ – a vector of household enabling variables and *X*_3i_ is a vector of village level variables and an error term *ϵ*_i_. All household socioeconomic variables, household enabling variables and village level variables are shown in Table 1. We show odds ratios of the association between the covariates and the decision to enrol in CBHI. To ascertain that the model is well fit, we first re-centre some variables to overcome multi-collinearity.^45^ We then show the Variance Inflation Factor statistic.

**Table 1:** Variable Type and Description

**Variable**	**Type and Variable Coding**
CBHI enrolment	Dummy: 1 = if a household was enrolled in CBHI, 0 = otherwise
Years in CBHI	Continuous: number of years in CBHI, ranging from 0 for the uninsured to 11 years
Child’s age (months)	Continuous: age in months
Mother’s age	Continuous: age in years
Birth weight	Continuous: weight in kilos
Household size	Continuous: number of people residing in the household
Parental (secondary) education	Dummy: 1 = if at least of the parents has a secondary education, 0 = none of the parents has a secondary education
Food adequacy	Dummy: 1 = if household states had enough food, 0 = if household states that food was not enough in the last 7 days
Household diet diversity score	Continuous: number of foods groups consumed in the last 7 days out of 12 food groups
Father employment = casual labourer	Dummy: 1 = father/husband’s employment is casual labour, 0 = father/husband’s employment not casual labour
Mother employment = casual labourer	Dummy: 1 = mother’s employment is casual labour, 0 = mother’s employment not casual labour
Health facility delivery	Dummy: 1 = if child delivered from a health facility, 0 = child not delivered in a health facility
Quintile 1 (poorest)	Categorical: divides a social economic wealth index into 5 categories: 1 = quintile 1 – poorest, 2 = quintile 2 – poorer, 3 = quintile 3 – average, 4 = quintile 4 – richer, 5 = quintile 5 – richest
Quintile 2 (poor)	
Quintile 3 (average)	
Quintile 4 (rich)	
Quintile 5 (richest)	
Has a neighbour in CBHI	Dummy: 1 = if one of the four immediate neighbours of a household is in CBHI, 0 = none of the neighbours is in CBHI
Access to information	Dummy: 1 = if the household had a television, or listened to radio daily or read a newspaper, 0 = household does not own a television, read a newspaper or listen to radio daily
Voluntary groups membership	Continuous: number of voluntary groups a household belongs/participates in
Perception index	Continuous: PCA generated index (first principal component) from 7 indices about perceptions on health insurance The index is made of 6 individual indices for premiums (5 questions), convenience of CBHI scheme (8 questions), benefits/financial protection (7 questions), quality of care (7 questions), management of the scheme (4 questions) and health beliefs (4 questions) and social influence (5 questions). Altogether, 42 questions in the index
Village health team	Dummy: 1 = if respondent has received any health advice from a community health worker in the last 12 months, 0 = otherwise
Know premiums	Dummy: 1 = if respondent knows insurance premiums per individual, 0 = otherwise
Health facility waiting time	Continuous: waiting time at health facility recorded in minutes
Size of burial groups	Continuous: number of households in a burial group a household belongs to
Number of burial groups in village	Continuous: number of burial groups in the village
Village has a school	Dummy: 1 = if village has a school, 0 = otherwise
Village has a health centre	Dummy: 1 = village has a health centre, 0 = otherwise
Trading trade	Dummy: 1 = if village main economic activity is retail trade, 0 = otherwise
Banana cultivating village	Dummy: 1 = if village main economic activity is banana cultivation, 0 = otherwise
Distance to health facility	Continuous: distance from village to commonly used health facility
Village altitude	Continuous: village altitude measured in metres above sea level

Abbreviations: CBHI, community-based health insurance; PCA, principal components analysis.

The decision to renew membership in CBHI is modelled in the form of the length of time households are insured. The more the years a household was in CBHI implies the number of annual renewing decisions taken by the households. As seen in the Figure, majority households (56%) are not in CBHI. These are therefore coded as zeros regarding the decision the renew insurance.

**Figure:**
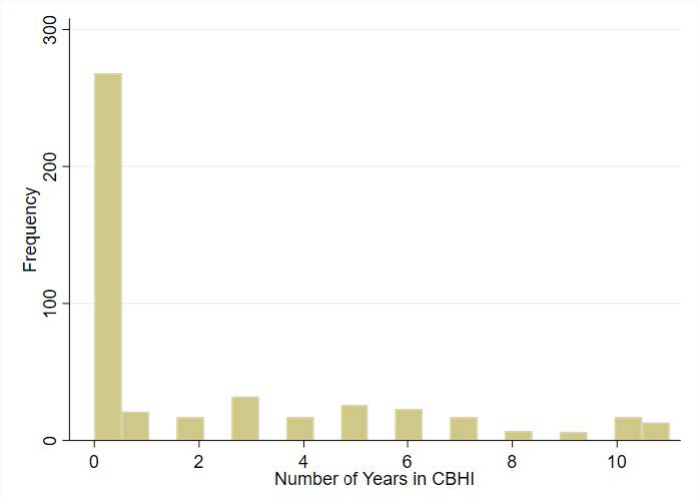
Number of Years in CBHI. Abbreviation: CBHI, community-based health insurance.

Because the outcome is a non-negative count outcome – years of participation in CBHI, a suitable model would be of a Poisson distribution, such as Poisson, Tobit, or negative binomial model. However, as the Figure shows, we are worried about excess zeros (over-dispersion) since more than half the sample does not renew participation. To model the determinants of renewing CBHI, we, therefore, use a zero-inflated negative binomial (ZINB) model. The ZINB model facilitates the estimation of a non-negative count outcome with possible over-dispersion better than other models for count outcomes.^46^ The ZINB model performs the inflation equation and an outcome equation. The inflation equation is a logistic estimation of the probability that the outcome is observed as a zero. After accounting for the excess zero in the model estimates the probability of the outcome.^47^ In order to show that the ZINB is the appropriate model over negative binomial model and other models of count outcomes, we show the Vuong test, which shows a significantly positive test statistic if the data is suitable for zero-inflated models. The basic model is then given as follows.

*Years*_i_ = *β*_0_*+β*_1_*X*_1i_*+ β*_2_*X*_2i_*+β*_3_*X*_3i_*+ϵ*_i_

Similar to determinants of CBHI enrolment status, renewing (*Years*_i_) is a function of vectors for household socioeconomic and demographic variables, household enabling variables and village covariates. In the results, we report incident rate ratios (IRRs) for renewing CBHI.

## Results

### Descriptive Results

Overall, 44% of the respondents were enrolled in CBHI and the average number of years of enrolment was 5 years. In Table 2, we detail summary statistics and the mean differences between CBHI and non-CBHI households, obtained through *t* tests. The average age for under-fives was 30.2 months while the average age for mothers was 30.2 years. 55.4% of the mothers had delivered their youngest child in a health facility. On average, birth weight was 3.1 kilograms but we observe substantial differences between CBHI and non-CBHI children.

**Table 2:** Descriptive Statistics – *T* Tests With Mean Differences in the Subgroups

	**Overall Mean**	**Mean Non-CBHI**	**Mean CBHI**	**Mean Difference**	***T*** ** Statistic**
Child’s age (months)	30.202	30.822	29.404	1.418	.318
Mother’s age	30.204	30.132	30.296	-0.164	.807
Birth weight	3.157	3.202	3.099	0.103	.026**
Household size	5.679	5.659	5.704	-0.045	.824
Catholic	0.504	0.383	0.660	-0.277	.001***
Parental (secondary) education	0.304	0.356	0.236	0.120	.005***
Food adequacy	0.534	0.510	0.567	-0.057	.224
Household diet diversity score	4.097	4.027	4.187	-0.160	.173
Father employment = casual labourer	0.356	0.299	0.414	-0.115	.010**
Mother employment = casual labourer	0.101	0.138	0.054	0.084	.003**
Health facility delivery	0.554	0.529	0.586	-0.057	.218
Wealth index (poorest)	-1.262	-1.291 (64)	-1.199 (30)	-0.092	.023**
Poorer	-0.777	-0.790 (53)	-0.759 (40)	-0.030	.231
Average	-0.299	-0.326 (48)	-0.273 (48)	-0.052	.077*
Richer	0.310	0.311 (42)	0.309 (53)	0.003	.961
Richest	2.211	2.370 (54)	1.943 (32)	0.427	.187
Access to information	0.599	0.548	0.665	-0.117	.011**
Has a neighbour in CBHI	0.692	0.521	0.911	-0.390	.001***
Voluntary groups membership	1.911	1.516	2.420	-0.904	.001***
Perception index	-0.000	-0.546	0.712	-1.247	.001***
Village health team	0.466	0.421	0.522	-0.101	.031**
Know premiums	0.528	0.241	0.897	-0.655	.001***
Health facility waiting time	88.621	71.540	110.581	-39.041	.001***
Size of burial groups	71.366	80.100	60.140	19.962	.001***
Number of burial groups in village	2.778	2.130	3.611	-1.481	.001***
Village has a school	0.528	0.628	0.399	0.229	.001***
Village has a health centre	0.401	0.460	0.325	0.135	.003***
Trading trade	0.366	0.391	0.335	0.056	.217
Banana cultivating village	0.261	0.249	0.276	-0.027	.515
Distance to health facility	11.239	12.646	9.429	3.217	.001***
Village altitude	1720.235	1671.336	1783.105	-111.769	.001***
*N*	464	261	203		

Abbreviation: CBHI, community-based health insurance. Significance levels for *P* values for * .10, ** .05, *** .01. For wealth index, the numbers of observations are 94, 92, 97, 95 and 86 for the poorest, poorer, average, richer and richest households, respectively. Observations for CBHI and non-CBHI households in parenthesis.

Birth weight was significantly lower in households with CBHI than those without. This might raise questions of adverse selection into insurance. However, as enrolment is group based and groups are independent of individual household preferences, it is highly doubted that households with low birth weight enrolled more than the rest. We also observe substantial differences in parental education and religion. CBHI households are more likely to be Catholic, which is the dominant religion in the area but are less likely to have secondary education. Men in insured households are likely to be employed in casual labour while only 5% of mothers in CBHI were casual labourers.

Socioeconomic welfare was assessed using principal components analysis (PCA)^48^ and combined 41 variables representing household asset holding, water and sanitation, agriculture and livestock assets and housing quality into a single index. On average, in terms of socioeconomic welfare, households in the richest quintile were almost 3 times better off than the poorest (bottom quintile) households. There is no substantial difference between the CBHI and non-CBHI in the richer households. Only in the poorest households, do observe substantial differences between the insured and non-insured households. Using PCA again, we follow Jehu-Appiah et al^22^ to develop a perception index. The perception index combines 42 Likert scale questions (see Supplementary file 1, Table S1) that elicit perceptions of 6 dimensions, namely; social influence, financial protection, premiums, health beliefs, management of schemes, the convenience of the scheme processes (such as enrolment requirements) and quality of care. A second PCA is then executed on these 7 indices to generate the first principal component as the perception index. To ascertain internal consistency of the indices developed, we provide the Cronbach’s alpha for the overall perceptions index and the 6 dimensions of perceptions in Table S2 in Supplementary file 1. Overall, we observe that households in CBHI have more positive perceptions than households not in CBHI.

As stated earlier, CBHI is accessed through burial groups. In principle, every household belongs to a burial group. Katabarwa^37^ has stated about their historical presence and Musau^35^ found that over 90% in south-western Uganda belonged to one. In this survey, virtually every household belonged to a burial group. We find that households in CHBI belonged to burial groups with an average of 60 households. Households that were not in CBHI were in generally larger burial groups averaging 80 households.

Villages can have several burial groups. We find households in CBHI belonged to villages with about 3.6 burial groups while non-CBHI belonged to villages with an average of 2.1 groups. We further indicate the differences in voluntary group membership, access to information and neighbourhood effects. Overall, households in CBHI belonged to more groups, had more access to information and had at least one neighbour in CBHI. We provide more descriptive results in Table 2.

### Empirical Results

### Determinants of Enrolment in Community-Based Health Insurance Scheme

Table 3 presents the results of a logistic regressions model for the determinants of enrolment in CBHI. We present the results in 3 models. Model one presents only household socioeconomic and demographic variables. Model 2 includes household enabling factors. These variables are not in direct control of a household but enhance the household’s capacity to participate in CBHI. The full model, Model 3 includes village covariates. First, we explore factors associated with reducing the odds of enrolment. We observe that households with older children are less likely to enrol as their odds of enrolment are lower by about 3% (odds ratio [OR]: 0.969, CI: 0.940-0.999) in the full model. The coefficient of the child’s age in months square is not statistically significant, implying that, as there is no evidence to suggest that, as children in households become older, household enrolment behaviour changes. Secondly, we observe that parental education is negatively associated with enrolment. Households whose at least one parent had a secondary education were less likely to enrol, with odds reduced by 60% (OR: 0.401, CI: 0.168-0.957) in model 2. However, once we control for village level covariates, the association though still negative, is no longer statistically significant. However, what is consistent in all models is the employment status of the women. We find that women employment in casual labour was negatively associated with enrolment. Odds were low by slightly over 71% (OR: 0.286, CI: 0.083-0.985).

**Table 3:** Logistic Regression Results for Determinants of Enrolling in CBHI^a^

**Variables**	**Model 1**			**Model 2**			**Model 3**		
	**OR**	***P*** ** Value**	**95 % CI**	**OR**	***P*** ** Value**	**95 % CI**	**OR**	***P*** ** Value**	**95 % CI**
Child age (months)	0.997	.653	0.981-1.012	0.973**	.041	0.947-0.999	0.969**	.040	0.940-0.999
Child age square	1.000	.474	0.999-1.001	1.000	.952	0.998-1.002	1.000	.606	0.998-1.001
Mother’s age	1.009	.665	0.969-1.051	0.999	.980	0.921-1.084	1.045	.367	0.949-1.151
Mother’s age square	1.000	.878	0.996-1.003	0.998	.566	0.993-1.004	0.997	.358	0.991-1.003
Birth weight	0.647*	.051	0.418-1.002	0.975	.924	0.573-1.659	0.746	.353	0.402-1.385
Household size	1.060	.363	0.935-1.203	0.978	.817	0.807-1.184	0.914	.456	0.721-1.158
Catholic	3.366***	.001	2.202-5.146	2.784***	.002	1.463-5.298	2.991**	.012	1.273-7.029
Parental (secondary) education	0.425***	.001	0.259-0.698	0.401**	.040	0.168-0.957	0.544	.223	0.205-1.447
Food adequacy	1.106	.671	0.695-1.759	1.131	.734	0.556-2.299	1.424	.414	0.610-3.324
Household diet diversity score	1.113	.277	0.918-1.349	0.984	.916	0.723-1.338	0.955	.786	0.684-1.333
Husband employment = casual	2.302***	.001	1.410-3.758	3.341***	.003	1.498-7.448	2.581**	.029	1.104-6.032
Mother employment = casual	0.281***	.001	0.138-0.574	0.273**	.012	0.099-0.749	0.286**	.047	0.083-0.985
Health facility delivery	1.342	.182	0.871-2.065	1.138	.718	0.565-2.292	1.097	.808	0.520-2.314
Wealth index (base: poorest)									
Poorer	1.371	.354	0.703-2.674	0.847	.748	0.307-2.336	0.742	.611	0.235-2.343
Average	2.075**	.043	1.022-4.212	3.533**	.023	1.194-10.455	2.615	.111	0.802-8.522
Rich	2.398**	.018	1.164-4.943	1.998	.248	0.618-6.463	1.301	.655	0.410-4.126
Richest	1.428	.373	0.652-3.126	4.102*	.059	0.948-17.762	3.790*	.081	0.847-16.950
Access to information	1.643**	.037	1.030-2.620	1.750	.152	0.813-3.768	1.880	.117	0.854-4.138
Has neighbour in CBHI				3.509***	.003	1.514-8.133	1.472	.508	0.468-4.625
Voluntary groups (number)				5.907***	.001	3.197-10.915	5.664***	.001	2.927-10.963
Voluntary groups square				0.528***	.001	0.380-0.734	0.612**	.012	0.416-0.899
Perception index				1.295**	.033	1.020-1.642	1.263*	.086	0.968-1.649
Village health team				1.896*	.079	0.929-3.871	1.440	.415	0.600-3.460
Know premiums				20.167***	.001	9.106-44.663	17.072***	.001	7.027-41.477
Waiting time				0.999	.683	0.996-1.003	0.999	.720	0.996-1.003
Burial group size				0.971***	.001	0.957-0.985	0.969***	.003	0.949-0.990
Burial groups in village (number)							1.208	.508	0.691-2.113
Village has school							0.653	.534	0.170-2.504
Village has health centre							1.197	.843	0.202-7.082
Trading village							0.314*	.092	0.082-1.208
Banana cultivating village							0.693	.768	0.061-7.910
Distance to health facility							0.826	.248	0.596-1.143
Distance square							1.033	.586	0.918-1.162
Village altitude							1.002	.808	0.989-1.015
Constant	0.185***	.000	0.090-0.378	0.009***	.000	0.002-0.046	0.026**	.016	0.001-0.501
Pseudo r-squared	0.147			0.615			0.662		
Variance inflation factor	1.92			2.06			3.02		
Observations	458			458			458		

Abbreviations: CBHI, community-based health insurance; OR, odds ratio. *** *P* < .01, ** *P* < .05, * *P* < 0.1. ^a^Outcome variable: CBHI status, 1 if insured, 0 otherwise.

We then turn to factors that enhance enrolment of households. As would be expected, there is a strong correlation between household wealth and enrolment status. Holding the poorest households as a comparison group, we find that as households improve in wealth, so do their odds of enrolment in CBHI. Average, richer and richest households were 2 to 4 times more likely to participate in CBHI. Once we control for household enabling variables we observed that households in average and richest classification were 3.5 times (OR: 3.533, CI: 1.194-10.455) and four times (OR: 4.102, CI: 0.948-17.762), respectively more likely to enrol. Once we control for additional village determinants, we observe that richest households were still close to 4 times (OR: 3.790, CI: 0.847-16.950) more likely to enrol compared to the poorest households. These unequal odds of enrolment based on socioeconomic welfare point to exclusion of the poorest despite the usefulness of existing informal risk-sharing mechanisms propagated through the burial groups as observed in this case study and elsewhere.^49,50^ Socioeconomic exclusion has been observed in studies in Ghana,^24^ though these and our findings here might differ from other studies that do not find a significant influence of socioeconomic status on enrolment.^51^

Husbands’ employment in casual work was associated with increasing the odds of enrolment by 2 to 3 times. In the full model, we observe that the odds of enrolment were higher by 2.6 times (OR: 2.581, CI: 1.104-6.032). In addition to the husband’s employment type, we also observe that belonging to the Catholic religion was associated with increasing the odds of participating in CBHI by up to 3.4 times. After the full model, we observe that being Catholic was associated with 3 times (OR: 2.991, CI: 1.273-7.029) more odds of enrolment compared to other religions. Regarding information, our measure assumes that owning a radio or television or access to newspapers frequently correctly measures access to information. We find that households with higher access to information had higher odds of enrolment (OR: 1.643; 95% CI: 1.030-2.620). However, while to coefficient generally increases, it is not significant when we control for group level enabling factors and village level covariates.

Model 2 includes several households enabling factors. These variables give us a host of social network and social connectivity proxies. We find that most of these indeed are associated with increased odds of enrolment. First, we observe that having a neighbour in CBHI increased odds of enrolment by 3.5 times (OR: 3.509, CI: 1.514-8.133). This association vanishes when we control for other community level variables. We find that belonging for more voluntary groups was associated with increasing enrolment by over 5 times (OR: 5.664, CI: 2.927-10.963). However, the relationship is non-linear in that enrolment reduces as a household participated in more voluntary groups. We find that what households know and what they perceive about insurance matters. Knowing premiums is a proxy of knowledge about CBHI processes, benefits, requirements and expectations. We find that knowing premiums was associated with increasing the odds of enrolment by up to between 20 times and 17 times in the full model (OR: 17.072, CI: 7.027-41.477). The perceptions index reflects how households generally think about health insurance in several dimensions. We find that holding more positive perceptions were associated with 26% to 30% higher odds of enrolment (OR: 1.263, CI: 0.968-1.649) in Model 3. However, belonging to a large burial group reduced enrolment by up to 3% (OR: 0.969, CI; 0.949-0.990). Finally, in Model 2, we observe that receiving advice from a community health worker was associated with increasing the odds of enrolment by close to 90% (OR: 1.896, CI: 0.929-3.871). However, the influence of community health workers reduces when we control for village level covariates in the full model.

### Determinants of Staying in Community-Based Health Insurance Scheme

The second major interest of this paper is to understand what influences households to renew participation in CBHI, especially in view of high dropouts recorded in similar CBHI schemes.^25,52^ After implementing ZINB models, we show results in Table 4, in 3 models for household socioeconomic and demographic variables, plus additional household enabling factors and the full model includes additional village covariates.

**Table 4:** ZINB Results of Determinants of Renewing CBHI^a^

**Variables**	**Model 1**			**Model 2**			**Model 3**		
	**IRR**	***P*** ** Value**	**95 % CI**	**IRR**	***P*** ** Value**	**95 % CI**	**IRR**	***P*** ** Value**	**95 % CI**
Child age (months)	1.007**	.013	1.001-1.012	1.007*	.092	0.999-1.014	1.006	.124	0.998-1.014
Child age square	1.000	.718	1.000-1.000	1.000	.677	0.999-1.000	1.000	.888	0.999-1.000
Mother’s age	1.049***	.001	1.032-1.066	1.042***	.001	1.019-1.065	1.045***	.001	1.021-1.069
Mother’s age square	0.997***	.001	0.996-0.998	0.996***	.001	0.994-0.998	0.996***	.001	0.994-0.998
Birth weight	1.067	.434	0.907-1.254	1.144	.235	0.916-1.427	1.103	.404	0.876-1.388
Household size	1.058**	.013	1.012-1.106	1.052	.131	0.985-1.125	1.034	.334	0.966-1.106
Catholic	1.134	.116	0.969-1.327	1.532***	.000	1.222-1.921	1.445***	.003	1.138-1.837
Parental (secondary) education	1.010	.916	0.845-1.206	0.845	.218	0.647-1.104	0.878	.331	0.674-1.142
Food adequacy	0.894	.193	0.756-1.058	0.906	.425	0.712-1.154	0.966	.790	0.750-1.244
Household diet diversity score	0.995	.890	0.931-1.064	0.981	.687	0.896-1.075	0.964	.451	0.877-1.060
Husband employment = casual	1.026	.758	0.870-1.210	1.141	.294	0.892-1.461	1.048	.711	0.819-1.340
Mother employment = casual	0.877	.397	0.646-1.189	0.532***	.003	0.351-0.806	0.641**	.042	0.417-0.985
Health facility delivery	0.995	.948	0.852-1.161	1.033	.771	0.829-1.288	1.024	.838	0.817-1.284
Wealth index (base: poorest)									
Poorer	1.037	.781	0.802-1.341	0.978	.906	0.679-1.409	0.854	.401	0.592-1.233
Average	1.201	.149	0.937-1.539	1.480**	.028	1.042-2.101	1.246	.229	0.870-1.785
Rich	1.108	.432	0.858-1.432	1.572**	.015	1.094-2.260	1.198	.344	0.824-1.740
Richest	1.199	.222	0.896-1.604	1.948***	.002	1.272-2.983	1.640**	.030	1.050-2.562
Access to information	1.062	.473	0.901-1.251	1.336**	.019	1.050-1.701	1.486***	.001	1.167-1.892
Has neighbour in CBHI				2.139***	.001	1.435-3.190	1.786***	.003	1.217-2.621
Voluntary groups (number)				2.189***	.001	1.776-2.699	2.260***	.001	1.835-2.783
Voluntary groups square				0.705***	.001	0.630-0.790	0.738***	.001	0.659-0.827
Perception index				0.965	.324	0.900-1.035	0.970	.416	0.902-1.044
Village health team				1.136	.234	0.921-1.401	1.008	.946	0.800-1.270
Know premiums				2.908***	.001	2.043-4.139	2.968***	.001	2.090-4.216
Waiting time				1.000	.708	0.999-1.001	1.000	.701	0.999-1.001
Burial group size				1.002	.396	0.997-1.007	1.007*	.066	1.000-1.014
Burial groups in village (number)							1.358***	.001	1.126-1.639
Village has school							1.527*	.095	0.929-2.508
Village has a health centre							1.117	.697	0.641-1.945
Trading village							0.347***	.001	0.210-0.574
Banana cultivating village							0.485*	.072	0.220-1.068
Distance to a health facility							0.811***	.001	0.718-0.916
Distance square							0.953**	.030	0.913-0.995
Village altitude							0.997*	.095	0.993-1.001
Constant	4.883***	.000	3.738-6.377	0.415***	.009	0.215-0.799	0.864	.774	0.319-2.340
Vuong (*P* value)	8.77 (0.001)			3.34 (0.001)			2.98 (0.001)		
Observations	458			458			458		

Abbreviations: CBHI, community-based health insurance; ZINB, zero-inflated negative binomial; IRR, incident rate ratio. *** *P* < .01, ** *P* < .05, * *P* < 0.1. ^a^Outcome variable: number of years in CBHI.

First, we observe that parental age plays an important role in renewing decisions. Households with older mothers are more likely to renew CBHI by an additional year (IRR: 1.045, CI: 1.021-1.069) however; renewing is less likely when as mothers get older as shown by the quadratic term of mother’s age. We find the households with older children were more likely to renew (IRR: 1.007, CI: 1.001-1.012), though the effect general reduced when we control for additional enabling and village covariates. Like the enrolment decisions, enrolled catholic households were more likely to renew CBHI, with an incident rate ranging from 45% to 53% (IRR: 1.445, CI: 1.138-1.837). Regarding socioeconomic status, we observe that richer households were more likely to renew membership. Controlling for socioeconomic and household enabling factors, average, rich and richest households were 1.5 to 1.9 times more likely to renew membership. Once we controlled for additional village covariates, we observed that the richest households were 1.6 times more likely to renew (IRR: 1.640, CI: 1.050-2.562).

The battery of enabling factors revealed similar effects on renewing as on enrolment. We observe that having a neighbour in CBHI was associated with increasing the likelihood of renewing CBHI by 2 times (IRR: 1.786, CI: 1.217-2.621) while belonging in an additional voluntary group was associated with an increased likelihood of renewing by up to 2.3 times (IRR: 2.260, CI: 1.835-2.783). However, households reduce renewing as they participate in more voluntary groups. Like enrolling decisions, households who knew the correct premiums levied were about 3 times (IRR: 2.968, CI: 2.090-4.216) more likely to renew CHBI membership. Belonging to a large burial group increased the likelihood of an insured household to renew membership by 0.7% (IRR: 1.007, CI: 1.000-1.014).

Regarding access to information, we find that households with more access to information had a higher likelihood of renewing membership, improved by close to 50% (IRR: 1.486; 95% CI: 1.167-1.892) in the full model. We find that like enrolment, households having a woman employed in casual labour were less likely to renew. In particular, the likelihood is reduced by between 53% and 64% (IRR: 0.641, CI: 0.417-0.985).

Many village level variables dampen renewing decisions. However, we find the households in villages with more burial groups were more likely to renew membership by 36% (IRR: 1.358, CI: 1.126-1.639). Likewise, residing in a village with a school as associated with a higher likelihood of renewing by up to 53% (IRR: 1.527, CI: 0.929-2.508). Finally, we highlight the influence of distance from health facilities. We find that an extra kilometre further from a health facility was associated with reducing enrolment likelihood by 29% (IRR: 0.811, CI: 0.718-0.916) and this association is linearly significant as shown by the quadratic term of distance from health facilities.

### Effect of Perceptions on Enrolment and Staying Insured 

Because behavioural change is embedded in community social structures, perceptions and beliefs are generally influential in the adoption of health behaviours. Perceptions about different aspects of health insurance generally play an important role in how individuals make decisions to enrol and utilise services.^22,53-56^, The perceptions presented here follow the classification of Jehu-Appiah and colleagues.^22^ In particular, we explore perceptions regarding management of the scheme, financial protection, health beliefs, social influence, the convenience of scheme processes, quality of health services and premiums. Due to collinearity in the indices, perceptions on scheme management and convenience of CBHI processes are not included in the regressions. As Jehu-Appiah et al^22^ have also done, we reverse the coding of premiums perceptions.

Table 5 shows the results of a logistic regression of the association of perceptions and enrolment decisions. In general, having more positive perceptions was associated with increasing the odds of enrolling in CBHI by 57% (OR: 1.568, CI: 1.390-1.769). Moreover, we were also interested in identifying which perceptions were more influential in decisions to enrol. All individual perceptions have a significant association with enrolment with perceptions on health beliefs having a negative association with enrolment behaviour (results are available upon request).

**Table 5:** Influence of Perceptions on Enrolment in CBHI

**Variables**	**Model 1**			**Model 2**		
	**OR**	***P*** ** Value**	**95% CI**	**OR**	***P*** ** Value**	**95% CI**
Overall perceptions index	1.568***	.001	1.390-1.769			
Perceptions on quality of care				1.151*	.076	0.986-1.345
Perceptions on health beliefs				0.965	.709	0.802-1.162
Perceptions on financial protection				0.955	.429	0.853-1.070
Perceptions on premiums				1.689***	.001	1.402-2.034
Perceptions on social influence				1.271***	.001	1.110-1.456
Constant	0.754***	.005	0.619-0.918	0.747***	.004	0.612-0.911
Observations	464			464		

Abbreviations: CBHI, community-based health insurance; OR, odds ratio. *** *P* < .01, ** *P* < .05, * *P* < 0.1.

Model 2 of Table 5 shows the association of individual perceptions and enrolment in a combined model. We observe that perceptions regarding the quality of care were associated with increasing the odds of enrolment by 15% (OR: 1.151, CI: 0.986-1.3459). Respondents who believed that the premiums were value for money and generally agreed with the ongoing premiums policy were more likely to be enrolled, having 69% higher odds of enrolment (OR: 1.689, CI: 1.402-2.034). Finally, we observe that enrolment decisions are not only a household choice but also households are influenced by people in their networks. We observe that feeling the social influence of leaders and relatives was associated with increasing the odds of enrolment by 27% (OR: 1.271, CI: 1.110-1.456). This finding makes important sense in view of how CBHI in south-western Uganda is linked with kin-associated burial groups.^37^

Furthermore, our analysis is interested in studying how perceptions influence decisions to renew CBHI membership. We implement a ZINB model due to the nature of our outcome – the number of years in CBHI. Results in Model 1 of Table 6 show that overall perceptions are not significant predictors of renewing decision, as seen in the main renewing model (in Table 4). More granular analysis of individual perceptions indicates that perceptions regarding social influence were significant predictors of renewing CBHI membership. In particular, respondents who had more influence from other people were 8.7% more likely to renew (OR: 1.087, CI: 1.002-1.178).

**Table 6:** Influence of Perceptions on Renewing CBHI

**Variables**	**Model 1**			**Model 2**		
	**IRR**	***P*** ** Value**	**95% CI**	**IRR**	***P*** ** Value**	**95% CI**
Overall perceptions index	0.995	.863	0.938-1.055			
Perceptions on quality of care				0.954	.238	0.882-1.032
Perceptions on health belief				1.048	.353	0.949-1.158
Perceptions on financial protection				0.995	.885	0.936-1.059
Perceptions on premiums				1.076	.156	0.972-1.190
Perceptions on social influence				1.087**	.044	1.002-1.178
Constant	5.028***	.000	4.525-5.587	4.790***	.001	4.281-5.359
Observations	464			464		

Abbreviations: CBHI, community-based health insurance; IRR, incident rate ratio. *** *P* < .01, ** *P* < .05, * *P* < 0.1.

The finding regarding other individual and overall perceptions might not imply that perceptions do not influence decisions of renewing CBHI but could rather indicate that once perceptions have been formed and initial enrolment decisions have been taken, households are more likely to keep in CBHI rather than update their perceptions in a way that negatively affects their enrolment status.

## Discussion and Policy Implications

### The Usefulness of Rural Employment Cash Flows 

We find different effects regarding the employment of men and women in casual labour. Non-farm employment, similar to casual labour in our study has been integral to rural employment and poverty reduction in Uganda.^57^ However, women and men participate in it differently. While only 10% of women in our sample were employed in this type of work, close to 36% of men were in casually employed. Casual employment is essential for village economies because it provides the type of cash flow required for burial groups in CBHI. Generally, all burial groups in the region and beyond exist to provide basic funeral insurance, however, they often go beyond only funeral insurance to provide credit services.^49,50^ Households with higher cash flows are therefore able to involve in lending, borrowing and saving to accumulate enough for premiums. Casual work, with higher cash flow, is appropriate for these demands. Moreover, the dynamics of casual work for women and men are different. While casual work might favour men due to mobility and opportunity to search for employment, women’s gender roles might imply that increasing their mobility (job search process) and uncertainty of employment in addition to traditional gender roles in rural areas can limit health utilisation behaviour. For instance, Morgan et al^58^ found that women’s workload limits the sustainable utilisation of maternal health services.

### Presence and Persistence of Socioeconomic Exclusion

The first one is that even in the presence of informal insurance systems which are supposedly inclusive,^50^ poorest households are still excluded. Rich households were 4 times more likely to enrol and 2 times more likely to renew CBHI than their poorest counterparts. The results are of pertinent interest to the government of Uganda, which is in the process of establishing a national health insurance programme. For the future of CBHI in particular and the national health insurance in general, it is recommended appropriate measures for inclusion are taken into consideration in the current planning processes. These measures might include premium waivers for the poor and progressive premiums for the better-off households as has been recently introduced in Rwanda.^59^ Another possible avenue of including the vulnerable population is taking CBHI within the broader spectrum of social protection programmes for the poor. In this case, social protection instruments such as cash transfers can supplement CBHI. Studies in Ethiopia have indicated that combining CBHI with other social protection programmes is beneficial for both health and socioeconomic outcomes.^60^ In addition, it might be important for development organisations supporting health insurance interventions to consider supporting the extremely poor households, either at a macro level through providing additional funds to insurance programmes^61,62^ or through direct identification and subsidising of the poor.^59^

### Social Connectivity and Access to Information Enhance Membership and Renewing

The influence of household enabling factors is noted. These factors are important proxies of social connectivity and social learning that takes place in health insurance programmes^63^ and other health interventions.^64,65^ In line with Liu et al,^63^ our findings suggest that households adopt and renew insurance through their social networks. An important network diffusion point in this study is burial societies in rural areas.^66^ Their usefulness in diffusing health information has been widely elaborated^67,68^ and it is our recommendation and CBHI promotion programme utilise them even more. Moreover, it is even important that the introduction of formal health insurance aims to build on existing informal risk management mechanism rather than bypass them. Bypassing them might erode social capital and eventual failure of the formalisation goals.^15^

The finding regarding the negative association of large burial groups can be explained by relating our case study scheme with the wider literature of group behaviour. A two-tailed condition for enrolment in this scheme is that for smaller burial groups of 30 or fewer households, all households are required to enrol while on the other tail, for larger burial groups, 60% of the households, which has to be higher than 30 households, are required for a group to enrol its members. With this condition in mind, the finding regarding burial group size aligns with other literature that large groups might portray less cooperation and more free-riding^69^ but also members might enjoy a higher utility from the wider risk-sharing networks,^70^ which might, in turn, reduce the propensity to formalise insurance by enrolling in CBHI.

Turning to access to information, we find that access to information increases the odds of enrolment as well as renewing membership in CBHI. Availability of information has been previously studied before in Ghana^71^ and Burkina Faso.^72,73^ In Burkina Faso, access to information was studied through an information, education and communication campaign while in Ghana it was studied through listening to radio, television or newspapers. Our findings. The findings are by and large mixed. The studies in Burkina Faso found that while insurance knowledge generally improved through access to information, it did not improve enrolment. However, the study in Ghana finds that exposure to all either radio or television or print media were all associated with increasing the odds of enrolment. Our findings are in line with this later Ghanaian study. However, while the current studies are focused on traditional media, there could be opportunities to utilising new types of media such as social media to spread information about insurance. Future studies could look into this issue.

### Perceptions Are Associated With Enrolment but not Renewing

Regarding the influence of perceptions, the study finds that households care about how the CBHI schemes are managed and this influences the decisions to enrol. Nevertheless, negative perceptions about premiums reduce the likelihood of both enrolling and renewing. These findings touch on the issue of trust, an underlying cause of failure in most CBHI schemes. Earlier work in Uganda found that low trust in schemes’ management was a major factor inhibiting enrolment. From a policy and implementation dimension, it is important to understand and consider how communities perceive CBHI. Trust and local buy-in might be achieved for instance by promoting more participation. Premiums and benefits packages, for instance, could be designed in more participatory ways. Understanding the importance of these perceptions is important for policy-makers and scheme managers in facilitating the development of easily saleable insurance interventions and benefits packages.

### Exploring the Potential of Faith-Based Health Providers in Insurance Expansion 

Finally, we would like to expound on the finding regarding higher enrolment and renewing of Catholic households. On average, just about half the households in our sample subscribed to the Catholic faith but over 66% of CBHI, households were Catholic. We do not have detailed data to look into why these households seem to insure more than others. However, we believe 2 mechanisms might be explored to increase future enrolment. First, larger group association through religious gatherings, helps in getting messages across to prospective insurance clients. Moreover, individuals might be more inclined to absorbing and acting of health messages from people of community respect such as religious leaders.^74,75^

Secondly, faith-based associations already play an important role in health service delivery in Uganda as well as providing health insurance options, especially to rural people. The scheme subject to this study is itself run under the auspices of the Uganda Protestant Medical Bureau and other faith-based medical bureaus run multiple facilities and insurance programmes.^76^ In establishing the national health insurance scheme, we recommend that policy-makers utilise these faith-based platforms in both marketing the scheme as well as maximising on their wide health infrastructure. Studies have shown that faith-based health providers in Uganda are intrinsically motivated to serve the poor^77,78^ hence inclusiveness might be achieved through these channels.

## Conclusion

We use a case study of a large CBHI scheme in south-western Uganda to shade more light on the reasons for enrolment and renewing of CBHI in rural Uganda. After logistic and ZINB regressions, we find that wealthier households were more likely to enrol in CBHI. Moreover, access to information and better social connectivity and husband’s employment in casual rural work were positively associated with enrolment decisions. In addition, wealthier households, households’ informal social support system assessed through membership burial groups and number of burial groups in the village was associated with renewing CBHI. Knowledge of CBHI assessed through knowledge of premiums strongly influenced both enrolment and renewing decisions. Moreover, improving perceptions about CBHI increases enrolment chances. Overall, by using this case study, the paper makes credible contributions to quantitatively understanding why households choose to enrol and renew in CBHI participation in rural Uganda. This is very crucial especially for the ongoing policy debates about a national health insurance programme.

## Acknowledgements

The authors acknowledge the support of all four research assistants that helped in household data collection. The support of the staff of Kisiizi hospital administration and the health insurance scheme, especially Dr. Francis Banya and Mr. Moses Mugume, is highly appreciated. The authors also highly appreciate assistance of the 5 research assistants that were involved in data collection.

## Ethical issues

The initial ethical review was carried out by the Center for Development Research (ZEF) research ethics committee at the University of Bonn, Bonn, Germany. Further reviews were conducted by the Mengo Hospital Research and Ethics Review Committee in Uganda. An ethical clearance certificate (Reference Number SS-39369) was issued by the Uganda National Council for Science and Technology. Informed verbal consent was obtained from all the survey respondents and the respective administrative leaders.

## Competing interests

Authors declare that they have no competing interests.

## Authors’ contributions

ENR led the conceptualisation of the study, design and acquisition of the data, statistical analysis, drafting the manuscript, and interpretation of the results. ECM supported drafting of the manuscript and data analysis. NN, NG, and JvB critically reviewed the manuscript. JvB and NG provided overall guidance and administration of the research project.

## Authors’ affiliations

^1^Institute for Food and Resource Economics (ILR), University of Bonn, Bonn, Germany. ^2^Department of Economics and Technological Change, Center for Development Research (ZEF), University of Bonn, Bonn, Germany. ^3^Department of Environmental and Public Health, Kabale University, Kabale, Uganda.

## Supplementary files


Supplementary file 1 contains Tables S1-S2.

## Key messages

**Implications for policy makers**

- Household’s socioeconomic welfare is strongly associated with While community-based health insurance (CBHI) enrolment and renewing decisions in rural Uganda.
- Social connectivity and access to information also predict household insurance status.
- It is important to consider community perceptions on health insurance to improve trust in insurance, enrolment and renewing.
- Burial groups in rural Uganda can act as critical entry points for formalising health insurance.

**Implications for public**

While community-based health insurance (CBHI) is expanding in many developing countries, with the targets of universal health coverage, enrolment remains low where programmes are voluntary in nature. Moreover, for those who enrol, dropping out is high. Understanding why households enrol and continue to renew their membership is central to achieving higher insurance coverage and ultimately universal coverage. Governments interested in reaching rural poor people with health insurance should consider maximising the potential of existing social support and informal insurance systems such as burial groups. Our research adds to a small body of literature on health insurance in Uganda and more broadly on renewing membership in insurance in developing countries.
