# Supplementary material for: Determinants of Enrolment and Renewing of Community-Based Health Insurance in Households With Under-5 Children in Rural South-Western Uganda
Source: Int J Health Policy Manag. 2019 Jul 6;8(10):593–606. doi: 10.15171/ijhpm.2019.49 (PMC6819630; doi:10.15171/ijhpm.2019.49)
Supplement: Supplementary file 1 — contains Tables S1-S2. [file ijhpm-8-593-s001.pdf]

Supplementary file 1

Table S1. Questions About Perceptions

The following questions were asked as 5-point Likert scale questions to generate 7 perception dimensions. The seven perception dimensions are then used in a principal components analysis to generate an overall perception index.

**Perceptions about premiums**	
The premiums/ enrolment fee charged on households should be reduced/ is too high	1=Strongly disagree<br>2=Disagree<br>3=Neither agree nor disagree (neutral)<br>4=Agree<br>5=Strongly agree
The scheme is more interested in making a profit than helping people like me	
I would rather use money for something else more economically beneficial than pay for insurance	
The co-payment is low and should be increased	
It happens often that a patient is denied his or her full entitlements as a fully enrolled member	
**Perceptions about the convenience of the CBHI scheme**	
The way insurance money is paid (through group leaders) is not safe	1=Strongly disagree<br>2=Disagree<br>3=Neither agree nor disagree (neutral)<br>4=Agree<br>5=Strongly agree
Group leaders prevent some people from joining the groups	
Group leadership should improve in order to encourage people to enrol	
Insurance outreach services are enough and we are satisfied	
Paying premiums through mobile money is convenient than paying through leaders	
The scheme managers listen to members feedback and use it to make the scheme better	
The window (insurance) opening hours are convenient all the time	
The process of getting registered is tedious	
**Perceptions about financial protection**	
We save money by being in insurance	1=Strongly disagree<br>2=Disagree
When in insurance, we do not have to borrow money from elsewhere to cater for health needs	

We are confident of seeking the best available healthcare without fearing for the its cost	3=Neither agree nor disagree (neutral)<br>4=Agree<br>5=Strongly agree
Being in the scheme means we do not have to sell our assets when sick	
Our income and savings are protected when we are in insurance	
There are other benefits of being insured on top of meeting our health care costs	
Joining insurance benefits me and my household	
**Perceptions about the quality of care for child illnesses**	
I am confident when I take my child to Kisiizi hospital for any health reason than other health facilities around	1=Strongly disagree<br>2=Disagree<br>3=Neither agree nor disagree (neutral)<br>4=Agree<br>5=Strongly agree
The scheme provides all necessary drugs when needed	
I believe that the drugs the scheme provides are of high quality	
The doctor/ nurse provides enough time to insured patients	
Doctors prefer cash paying people than insured people	
The scheme makes patients pay for most of the drugs	
Doctors and nurses are very caring and do not abuse us.	
**Perceptions about the attitudes of providers**	
The management of the scheme has the skill to manage the scheme very well	1=Strongly disagree<br>2=Disagree<br>3=Neither agree nor disagree (neutral)<br>4=Agree<br>5=Strongly agree
Staff are the scheme are sometimes rude and make people shy from approaching them for help	
Staff are always helpful and making sure that patients get the best quality care	
Other hospital staff (such as medical professionals, accounts people and others) treat all patients with equal respect	
**Perceptions about social influence from other community members**	
We learn from our neighbours about the things we do such as which community groups to join	1=Strongly disagree<br>2=Disagree
Village opinion leaders influence us about the programmes we enrol in, such as insurance	

Our friends and other extended family members influence our decision to enrol in insurance	3=Neither agree nor disagree (neutral)<br>4=Agree<br>5=Strongly agree
Enrolling in insurance as an individual household would be better than the current condition to belong in a group	
The experiences of other community members with insurance affects our decision to enrol in insurance	
**Perceptions about health beliefs**	
Every village (or parish) requires at least one traditional birth attendant	
Buying health insurance is bad luck, it implies that we are positively embracing/ welcoming sickness in our household	
Health is a matter in God's hands and insurance cannot help me deal with it.	
Insecticide treated mosquito nets make sleeping uncomfortable	

Table S2. Cronbach's Alpha Measured of Internal Reliability of the Items in Principal Components Analysis

Perception measurement	Cronbach's alpha
Perceptions about premiums (5 items)	0.3691
Perceptions about scheme convinience (8 items)	0.6731
Perceptions about financial protection (7 items)	0.8517
Perceptions about quality of care (7 items)	0.6295
Perception about scheme management (4 items)	0.7109
Perceptions about social influence (5 items)	0.6722
Perceptions about health beliefs (4 items)	0.2966
Overall perception index (7 items)	0.7558
